# Emergency irradiation of 3.4Gy/2f in pineal gland germinoma patients with symptomatic hydrocephalus

**DOI:** 10.1186/s41016-019-0160-0

**Published:** 2019-06-03

**Authors:** Bo Li, Youqi Li, Chunde Li, Yanwei Liu, Shuai Liu, Xiaoguang Qiu, Shiqi Luo

**Affiliations:** 1 Department of Radiation Oncology, Beijing Tiantan Hospital, Capital Medical University, No. 119 South 4th Ring West Road, Fengtai District, Beijing, 100070 China; 2 Department of Neurosurgery, Beijing Tiantan Hospital, Capital Medical University, No. 119 South 4th Ring West Road, Fengtai District, Beijing, 100070 China

**Keywords:** Germinoma, β-HCG, Chemotherapy, Radiotherapy, Hydrocephalus, Visual field defect

## Abstract

**Background:**

Surgical interventions including ventriculostomy and ventriculo-peritoneal shunt were usually administrated in pineal germ cell tumor patients with symptomatic hydrocephalus. Considering higher sensitivity of germinoma to anti-tumor therapy, we explored emergency irradiation as non-invasive measure in this situation.

**Methods:**

Data of 35 germinoma patients with symptomatic hydrocephalus who received emergency irradiation of 3.4 Gy/2f were studied retrospectively. The maximum width of frontal horn and the minimum width of trunk of corpus callosum (TCC) were measured to evaluate hydrocephalus changing. Besides, mean deviation (MD) of Humphrey perimetry was employed to evaluate visual field defect. Correlations between hydrocephalus changing and clinical factors, including age, percentage of tumor regression, radiographic re-evaluation interval, and serum beta-human chorionic gonadotropin (β-HCG) level, were analyzed.

**Results:**

The median maximum diameter and volume of pineal lesions was 27 mm (range 10–55 mm) and 6.5cm^3^ (range 0.4–74.1 cm^3^), respectively. At median 8 days after irradiation, the median percentage of tumor remission was 55% (range 10–100%). The median maximum width of FN and the median minimum width of TCC were 11.6 mm and 39.0 mm, and 8.0 mm and 31.4 mm, before and after irradiation, respectively. The improvement of both parameters reached significant level (*p* < 0.001). However, none clinical factor was found to have correlation with their improvement. In 14 patients with paired data of pre- and post-irradiation MD, its change did not reach the significant level for both eyes. All patients successfully received subsequent chemoradiotherapy without surgical intervention.

**Conclusions:**

Emergency irradiation of 3.4 Gy/2f was an effective non-invasive measure to relief hydrocephalus in pineal germinoma patients.

## Background

Intracranial germ cell tumors (iGCTs) are rare malignancies that are mostly identified in children and adolescence. In Western counties, it accounts for 3–5% of primary central nervous system carcinomas of children and young adults, while that number is 11–15% in Asian populations [1-3]. Generally, iGCTs are segregated into germinomas and non-germinomatous germ cell tumors (NGGCTs) in most classifications. Patients with germinoma had favorable prognosis, with cure rate of more than 90% [4, 5].

Pineal gland is one of the most common involved area, which accounts for 50–60% of iGCTs in some reports [6]. iGCTs involving the pineal gland often cause increased intracranial pressure as a result of aqueduct obstruction and may also cause Parinaud’s syndrome (vertical gaze palsy and a pseudo-Argyll-Robertson pupil) in up to 50% of cases [7]. Clinically, ventriculo-peritoneal shunt was usually implanted to relive symptoms of obstructive hydrocephalus and pave the way for the subsequent anti-tumor procedure. In addition, endoscopic third ventriculostomy was alternative which allows tumor biopsy and improvement of hydrocephalus simultaneously. However, invasive techniques as they are, the complications such as catheter obstruction, broken catheter, and infection are of concerns. Furthermore, permanent catheter-carrying also has a negative impact on the patient’s quality of life. Nowadays, a biopsy is generally not attempted in patients with typical radiographic findings and elevated tumor markers such as beta-human chorionic gonadotropin (β-HCG) and alpha fetoprotein (AFP). Besides, primary surgery is not recommended for germinomas as they are sensitive to chemotherapy and radiotherapy, and the risk of surgical complications usually outweighs any potential benefit [8]. Thus, in our hospital, emergency irradiation of 3.4 Gy/2f was employed in patients who were diagnosed as pineal gland germinoma with symptomatic hydrocephalus, which intend to avoid invasive procedure. Here we present the results.

## Methods

### Patient inclusion

Medical records of patients who were diagnosed as germinoma at Beijing Tiantan Hospital affiliated to Capital Medical University between January 2014 and December 2017 were screened. The inclusion criteria were (1) age < 30 years; (2) primary lesion located in pineal gland region; (3) on magnetic resonance imaging, the lesion was isointense on T1-weighted images and enhanced homogeneously; (4) at least one of serum or cerebral spinal fluid (CSF) beta-human chorionic gonadotropin (β-HCG) was elevated mildly (5.0 IU/L < β-HCG < 100 IU/L); and (5) emergency irradiation was administrated due to hydrocephalus. Exclusion criteria were (1) the primary lesion located other than pineal gland region, (2) alpha fetoprotein (AFP) was elevated, and (3) ventriculo-peritoneal or ventriculostomy was the initial treatment modality. Clinical data of eligible patients were obtained from institutional archive and analyzed retrospectively. This study was approved by the institutional review board.

### Treatment strategy

Before treatment, all patients underwent a baseline evaluation, including a medical history, a general physical examination, complete blood count, serum chemistry, serum and/or CSF tumor markers (β-HCG and APF), and radiographic examinations (plain computed tomography scan, enhanced-contrast magnetic resonance image). Visual examination was recommended in patients with visual complaints. If symptoms of high cranial pressure could be relieved by mannitol infusion at 12-h intervals or less and there is no indication for surgical interventions, emergency irradiation of 3.4 Gy/2f was employed to the primary lesions. About a week later, baseline evaluations were repeated and spinal MRI was conducted. For patients with tumor regression, 2 cycles of platinum-based chemotherapy were administrated followed by radiotherapy. Or else, surgery would be considered. Radiotherapy consisted of whole brain radiotherapy (WBRT) with a dose of 24–30 Gy and a boost to the primary lesions. The total prescription dose was up to 50 Gy for patients who had complete remission (CR) to chemotherapy, and up to 55–60 Gy for those with less than CR. Patients with disseminated disease or older than 16 years also received cranial spinal irradiation (CSI) to the entire cranial contents and spinal axis. After the completion of radiotherapy, up to 4 cycles of platinum-based chemotherapy was recommended. Then, routine follow-up was repeated every 3 months for the first 2 years, and every 6 months for the next 3 years.

### Response evaluation

**Fig. 1:**
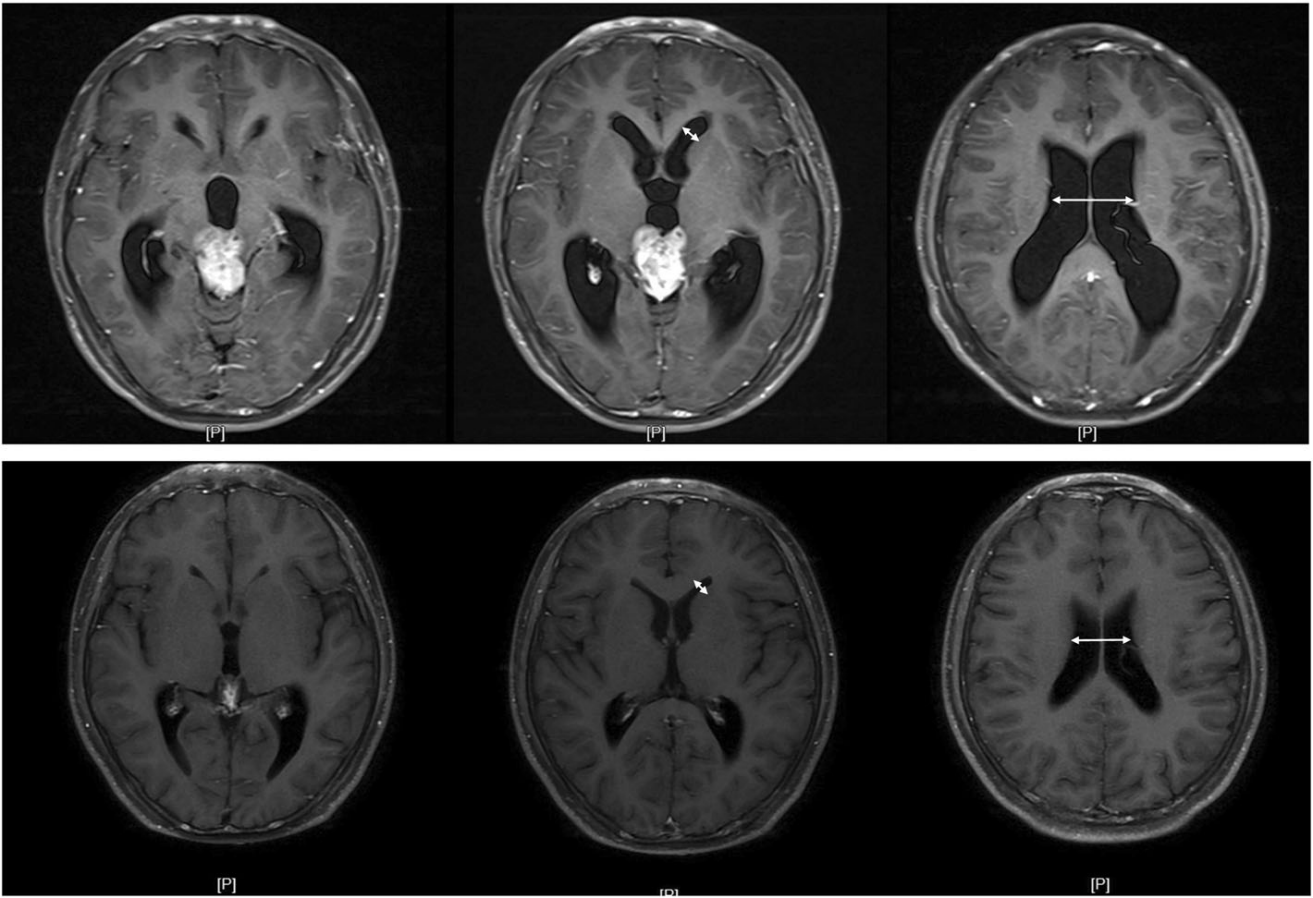
The maximum width of frontal horn (FH) and the minimum width of trunk of corpus callosum (TCC) (white arrows) on T1-weighted enhanced-contrast images were measured to evaluate hydrocephalus changing before (upper axial images) and after (lower axial images) emergency irradiation

The evaluation of tumor response was based on enhanced-contrast MRI and the two-dimensional RANO (Response Assessment in Neuro-oncology) criteria by enhancing the tumor area (the maximum cross-sectional enhancing diameters) as the primary tumor measure. Furthermore, the maximum width of frontal horn (FH) and the minimum width of trunk of corpus callosum (TCC) on T1-weighted enhanced-contrast images were measured to evaluate hydrocephalus changing (Fig. 1).

### Statistical analysis

IBM SPSS Statistics 19.0 software was used for the data analysis. Mann-Whitney *U* test was used for continuous variables. Chi-square test was used for category variables. Spearman test was used to analyze the correlation between hydrocephalus changing and clinical factors such as age at diagnosis (years), radiographic re-evaluation interval (days), the percent of tumor regression, the serum level of β-HCG. A *p* value less than 0.05 was used as the criterion of statistical significance, and all statistical tests were two-sided.

## Results

### Patients’ characteristics

There are 35 patients diagnosed from October 2014 to September 2017 included in this study. Patients’ characteristics were summarized in Table 1. In our series, 85.7% (30/35) of patients were male and 14.3% (5/35) were female. The median age was 16 years (range 5–29 years). Altogether, 65.7% (23/35) of patients had their primary lesions located in the pineal gland region. Twenty percent (7/35) of patients were bi-focal, who had lesions both in the pineal gland and suprasellar region. The other 5 patients had disseminated disease in addition to the primary lesions of the pineal gland. The median maximum diameter of pineal gland lesions was 27 mm (range 10–55 mm) and the median volume of pineal gland lesions was 6.5 cm^3^ (range 0.4–74.1 cm^3^), respectively.

**Table 1:** Patient characteristics

Characteristics	*N* = 35	Percent
Median age	16 years	Range 5–31 years
Gender		
Male	30	85.7
Female	5	14.3
Location		
Pineal gland region	23	65.7
Sellar/suprasellar and pineal gland region	7	20.0
Pineal gland region with ventricular dissemination	2	5.7
Sellar/suprasellar and pineal gland region with ventricular dissemination	3	8.6
β-HCG elevation	25.3 IU/L	Range 7.2–75.7 IU/L
Median maximum diameter of pineal gland lesions	27 mm	Range 10–55 mm
Median volume of pineal gland lesions	6.5 cm^3^	Range 0.4–74.1 cm^3^
Symptoms^a^		
Headache, nausea, vomiting	28	80.0
Visual complaints	19	54.3
Adipsic diabetes insipidus	8	22.8
Syrigmus	5	14.3
Drossy	5	14.3
Hypomnesia	4	11.4

*Abbreviations*: *β-HCG* beta-human chorionic gonadotropin

^a^One patient may have more than one symptom

In terms of symptoms, those correlated with high cranial pressure were the most common, which were presented in 80% of patients (28/35). Visual complaints ranked the second, in which visual acuity decline was seen in 4 patients and diplopia was seen in 15 patients. In 4 patients who complained of visual acuity decline, 2 had suprasellar lesions. Although adipsic diabetes insipidus was not the most common symptom, it was presented in 80% (8/10) of patients who accompanied suprasellar lesions. Other symptoms included syrigmus in 5, drossy in 5 and hypomnesia in 4.

### Treatment results

The median radiographic re-evaluation interval was 8 days (range 4–16 days). And the median percentage of tumor remission was 55% (range 10–100%), which 62.9% (22/35) of patients had tumor regression ≥ 50% (including complete remission in 1 patient). No toxicities were observed. Subsequently, 26 patients received 2 cycles of chemotherapy and 7 received 1 cycle. Before radiotherapy, CR was achieved in 30 patients and 3 patients still had residue disease (< 0.5 cm). Two patients had no chemotherapy prior to radiotherapy because of alanine transaminase elevation (*n* = 1) or left ventricle dilation (n = 1). In terms of radiotherapy, 14 patients received WBRT plus primary boost, 21 patients received CSI. The median prescription dose was 50 Gy (range 32–60 Gy). The median follow-up period of our series was 20 months (range 6–41 months). No one relapsed or died by far.

### Hydrocephalus

The median maximum width of FN and the median minimum width of TCC before emergency irradiation were 11.6 mm (range 3.0–23.1 mm) and 39.0 mm (range 19.4–56.4 mm), respectively. While that number was 8.0 mm (range 2.0–15.0 mm) and 31.4 mm (range 12.0–49.3 mm), respectively at the time of radiographic re-evaluation. Statistical analysis found the improvement of these two parameters reached a significant level (Table 2). However, none of the clinical factors such as age at diagnosis (years), radiographic re-evaluation interval (days), the percent of tumor regression, and the serum level of β-HCG was found to have a correlation with the improvement of these two parameters (Table 3). No patient received surgical intervention because of hydrocephalus during the treatment or follow-up.

**Table 2:** The changing of hydrocephalus and visual field before and after emergency irradiation

Parameters	Pre-emergency-irradiation	Post-emergency-irradiation	*P* value
Hydrocephalus improvement (mm)			
Maximum width of FN	11.6 (range 3.0 to 23.1)	8.0 (range 2.0 to 15.0)	< 0.001
Minimum width of TCC	39.0 (range 19.4 to 56.4)	31.4 (range 12.0 to 49.3)	< 0.001
Median MD (*n* = 14)			
Right eyes	−4.6(range − 10.1 to − 0.9)	−2.2(range − 7.3 to − 0.4)	0.18
Left eyes	−3.4(range − 10.8 to − 1.4)	−3.0(range − 14.5 to − 0.7)	0.87

*Abbreviations*: *FN* frontal horn, *TCC* trunk of corpus callosum, *MD* mean deviation

**Table 3:** The correlation between hydrocephalus improvement and clinical factors

	Age at diagnosis		Extend of tumor regression		Radiographic re-evaluation interval		β-HCG	
	Correlation coefficient	*P* value	Correlation coefficient	*P* value	Correlation coefficient	*P* valve	Correlation coefficient	*P* valve
Improvement of maximum width of FN	− 0.192	0.30	0.246	0.18	0.021	0.91	0.223	0.22
Improvement of minimum width of TCC	− 0.204	0.27	0.214	0.25	− 0.063	0.74	0.319	0.081

*Abbreviations*: *FN* frontal horn, *TCC* trunk of corpus callosum, *β-HC* beta-human chorionic gonadotropin

### Visual examination

Altogether, 14 patients received Humphrey perimetry examination before and after emergency irradiation. The median deviation (MD) of right eyes and left eyes before emergency irradiation was − 4.6 (range − 10.1 to − 0.9) and − 3.4 (range − 10.8 to − 1.4), respectively. While those numbers were − 2.2 (range − 7.3 to − 0.4) and − 3.0 (range − 14.5 to − 0.7), respectively after emergency irradiation (at the time of radiographic re-evaluation). However, the difference of MD changing before and after irradiation did not reach the significant level (Table 2). The best-corrected visual acuity (BCVA) was available in 5 patients before emergency irradiation. In 3 patients with sellar/suprasellar region involvement, the BCVA of both eyes (right/left) were 0.4/0.5, 0.12/0.05, and 0.04/0.1, respectively. While in 2 patients with pineal gland lesions only, those numbers were 0.8/0.8 and 1.0/1.0, respectively. Unfortunately, only 1 patient had paired BCVA which was improved from 0.04 to 0.4 on the right and from 0.1 to 0.25 on the left.

## Discussion

In our study, we explore the efficacy of emergency irradiation of 3.4 Gy/2f in patients who were diagnosed as pineal gland germinoma with symptomatic hydrocephalus. As a result, the hydrocephalus improved significantly and no one received surgical intervention because of hydrocephalus during the treatment.

In iGCTs patients involving pineal gland region, obstructive hydrocephalus was a common complication. Surgical interventions such as ventriculo-peritoneal shunt or endoscopic third ventriculostomy were usually considered to not only relief the symptoms related to hydrocephalus, but also provide an opportunity to obtain pathological diagnosis. However, with typical radiographic findings and elevated tumor markers, clinical diagnosis of iGCTs could also be established [9]. Furthermore, in patients with germinoma, surgical resection could be omitted from initial treatment since they are extremely sensitive to chemotherapy and radiotherapy [8]. Thus, it is reasonable that invasive measures could be spared if proper treatment modality was taken in pineal gland germinoma patients with hydrocephalus. Hence, in our study, emergency irradiation of 3.4 Gy/2f was employed as an initial treatment modality in patients with isointense lesions and β-HCG mild elevation, which is essential for the diagnosis of germinoma. As a result, the efficacy was satisfactory since the tumor regression was seen in all patients. And around 60% of patients had their tumor shrunk more than 50%, including complete remission in one patient. Moreover, the hydrocephalus which was evaluated by the maximum width of FN and the minimum width of TCC also improved significantly and surgical intervention was exempted for these population.

The successful administration of this technique relied not only on accurate diagnosis, but also proper evaluation of the severity of the hydrocephalus. In our experience, only those with “mild” hydrocephalus may benefit from the non-invasive modality. However, in clinical practice, there are no consensus criteria on how to classify the severity of hydrocephalus by far. Although the extent of ventricular dilation, which was evaluated by the maximum width of FN and the minimum width of TCC in our study, seems to be the candidate parameter, it had neither correlation with the pre-irradiation volume nor the percentage of tumor regression of pineal gland lesions. Besides, symptoms are not usually in accordance with the extent of ventricular dilation, since the hydrocephalus development speed and patient tolerance were also correlated. Thus, in our study, the frequency of mannitol infusion was adopted as a criterion to evaluate the severity of hydrocephalus. Patients who can achieve symptom relief were potential candidates for the non-invasive measures. Otherwise, surgical intervention is still the effective way to not only relieve the symptoms but also pave the way for the subsequent treatment. Hence, full communication with surgeons is extremely important until the reliable criterion was established to better define beneficial populations.

In addition to radiotherapy, chemotherapy was another initial treatment option in patients with germinoma. Many studies have found, the addition of induction chemotherapy could make radiation dose and/or field reduction possible without compromising long-term survival in patients with localized lesions [4, 10, 11]. However, in pineal gland iGCTs with hydrocephalus, a dilemma might arise if chemotherapy was employed initially. According to Children’s Oncology Group (COG) criteria, NGGCTs are defined if AFP is above the institutional upper limit and/or β-HCG is higher than 100 IU/L. In Europe, if AFP is higher than 25 ng/ml and/or β-HCG higher than 50 IU/L, the diagnosis of NGGCTs should be considered. In some clinical trials, 50 IU/L of β-HCG was also used as a cut-off for germinoma patient recruitment [4, 10]. However, patients with mild elevation of β-HCG only were just considered as “germinoma,” NGGCTs components, which were less sensitive to anti-tumor therapy, could not be ruled out completely [9, 12]. As a result, it is problematic if hydrocephalus did not improve satisfactorily after full-dose chemotherapy delivering and surgical intervention was inevitable, especially during the peri-bone-marrow-suppression period. Local therapy as it is, the emergency irradiation we employed has limited influence on potential surgical interventions. Furthermore, the following induction chemotherapy provided a second chance for tumor sensitivity evaluation, which is important for the diagnosis of germinoma without histology support.

According to the study conducted by Allen et al., β-HCG elevation, either in the serum or CSF, was reported in only about 40% of germinoma patients [13]. Most patients were negative for tumor makers and histology examination was recommended by most oncologists [14]. However, in clinical practice, there are still difficulties in obtaining histological diagnosis in all tumor maker-negative patients. Diagnostic radiotherapy, which was firstly reported by Japanese authors, was also employed as an alternative measure in many centers, including ours [15, 16]. Different from the dose of 20 Gy/10f reported in a Japanese study, in our hospital, a lower dose was delivered due to concerns of potential toxicities and misdiagnosis. The results from our preliminary studies conducted in 2006 showed that 10 Gy/5f was comparable to 20 Gy/10f in terms of tumor regression since over 90% of lesions shrunk more than 50% [17, 18]. In recent years, 3.4 Gy/2f was recruited in our clinical practice, not only as diagnostic radiotherapy regimen, but also as a measure in an emergency situation such as hydrocephalus. Although the tumor regression was less effective compared with the higher dose regimen, we found the hydrocephalus improvement has no correlation with the extent of tumor regression. While as a diagnostic measure, 3.4 Gy/2f was applicable since all patients in our series who were considered as germinoma had their lesions regressed. Furthermore, the potential side effects of 3.4 Gy/2f might be minimum when the diagnosis was adjusted in poor responders.

Visual complaints were also common in patients with iGCTs, including visual acuity decline, visual field defect, ocular movement disturbance, etc. However, there are differences among patients involved different intracranial locations. In patients with pineal gland lesions, diplopia seems the most common, while visual acuity and field impairment occurred more often when sellar/suparsellar region involved. In a retrospective study including 28 iGCTs, 12 (42.8%) patients reported visual disturbances [19]. In 7 patients that suffered from visual acuity loss, 5 (71.4%) had suprasellar involvement. In 8 patients that complained of hemianopsia, 7 (87.5%) had suprasellar involvement. While in 3 patients with diplopia, 2 had pineal gland lesion only. In another study that recruited 16 sellar/suparsellar germinoma patients, 8 (50%) had visual acuity problems [20]. As it was shown in our study, 15 (78.9%) patients had diplopia among 19 patients with visual complaints. Otherwise, visual acuity decline seems more common and severe in patients with sellar/suprasellar region involvement. In terms of visual field defect, data of Humphrey perimetry showed, most patients had mild MD decrease and its improvement did not reach the significant level along with the hydrocephalus improvement. Hence, ocular movement evaluation should be recommended preferentially in patients with pineal gland lesions. In addition, in patients with sellar/suprasellar lesions, visual acuity and visual field examinations should be considered, either.

## Conclusions

Obstructive hydrocephalus was a common complication in patients with pineal gland iGCTs. In our study, emergency irradiation of 3.4 Gy/2f was employed as a treatment modality in this situation. As a result, it has proved to be an effective non-invasive measure to relieve hydrocephalus in patients who were considered as pineal gland germinoma. Further study is needed to establish criteria evaluating the severity of hydrocephalus for beneficial population screening.

## Abbreviations

AFP: Alpha fetoprotein
BCVA: Best-corrected visual acuity
COG: Children’s Oncology Group
CR: Complete remission
CSF: Cerebral spinal fluid
CSI: Cranial spinal irradiation
FH: Frontal horn
iGCTs: intracranial germ cell tumors
MD: Mean deviation
NGGCTs: Non-germinomatous germ cell tumors
RANO: Response Assessment in Neuro-oncology
TCC: Trunk of corpus callosum
WBRT: Whole brain radiotherapy
β-HCG: Beta-human chorionic gonadotropin
